# The Disinfection Characteristics of Ebola Virus Outbreak Variants

**DOI:** 10.1038/srep38293

**Published:** 2016-12-02

**Authors:** Bradley W. M. Cook, Todd A. Cutts, Aidan M. Nikiforuk, Anders Leung, Darwyn Kobasa, Steven S. Theriault

**Affiliations:** 1 Applied Biosafety Research Program, Public Health Agency of Canada, Winnipeg, Manitoba, Canada; 2 Department of Microbiology University of Manitoba, Winnipeg, Manitoba, Canada; 3 High Containment Respiratory Viruses Group, Special Pathogens Program, Public Health Agency of Canada, Winnipeg, Manitoba, Canada; 4 Department of Medical Microbiology, University of Manitoba, Winnipeg, Manitoba, Canada

## Abstract

The recent Ebola virus outbreak in West Africa has forced experts to re-evaluate their understanding of how to best disinfect areas contaminated with infectious bodily fluids. Recent research has found that Ebola virus remains viable in blood for 7–10 days making appropriate disinfection crucial to infection control. We sought to determine if the three most important outbreak variants of *Zaire ebolavirus* (Mayinga, Kikwit and Makona) exhibit separate phenotypes when challenged with a range of sodium hypochlorite (NaOCl) concentrations or 70% ethanol (EtOH) at average West African temperature. The time dependent killing of Ebola virus was evaluated by measuring infectious virus and viral RNA (vRNA), to determine if RNA detection is a viable method for decontamination measurement in areas without high containment laboratory access. Makona was less susceptible to weaker concentrations of NaOCl (0.05 and 0.1%) than Mayinga and Kikwit. At the recommended concentration of NaOCl (≥0.5%) all of the variants were inert after 5 minutes of contact time. Similarly, all variants were inactivated by 70% EtOH after 2.5 minutes, only Makona was detected at 1 minute. In multiple instances, high amounts of vRNA was detected in the absence of infectious virus, suggesting that it does not serve as an accurate measure of remaining infectivity after cleansing.

The *filoviridae* have two genera, *Marburgvirus* and *Ebolavirus* with the latter consisting of five species: *Sudan ebolavirus* (SEBOV), *Bundabugyo ebolavirus* (BEBOV), *Tai Forest ebolavirus* (TEBOV), *Reston ebolavirus* (REBOV) and *Zaire ebolavirus* (ZEBOV). The ZEBOV subtype has proved the most deadly to the population of Central and West Africa with three major outbreak variants. The 1976 outbreak variant Mayinga was responsible for 380 cases with 218 fatalities (88% case-fatality rate (CFR)), and the 1995 Kikwit variant caused 315 cases with 250 mortalities (81% CFR)^1^. These previous outbreaks starkly contrast to the 2013–2016 West African outbreak of the Makona variant which had 28, 616 confirmed cases and 11, 310 deaths^2^. Furthermore, there have been multiple flare-ups associated with exposure to infectious bodily fluids from Ebola survivors, most notably semen^3^,^4^,^5^.

Published environmental studies on Ebola have been rather limited. The severity of the West African outbreak has led to a surge in investigations. These efforts have uncovered that Ebola virus can remain viable for significantly lengthy periods of time on multiple surfaces found in the environment and as a component of personal protective equipment (PPE)^6^,^7^,^8^,^9^,^10^. Conditions including the type of surface material, temperature and humidity greatly influence virion stability. These observations highlight the necessity for disinfection of Ebola contaminated surfaces, as patients with severe Ebola virus disease (EVD) may excrete nearly 8 litres of infectious bodily fluids per day^11^. When combined with the waste generated from the cleaning of Ebola treatment centers (ETC), the estimated volume of liquid waste entering a disposal tank totals 300 litres a day per patient^12^,^13^. If inadequate disinfection methods are used than the liquid waste generated from an ETC can contain infectious virus and pose a significant risk of environmental exposure. Although Ebola has not yet been demonstrated to survive in grey water, it does persist in some liquid mediums nearly as long as on solid materials.

Ebola virus can persist in dried human/non-human primate blood 7–10 days at West African climate conditions (28 °C/90% relative humidity (RH))^7^,^8^ and the Makona variant remained viable longer than the Mayinga variant regardless of temperature and RH conditions^8^. This suggests that the Makona variant may behave quite differently with respect to inactivation or decontamination methods, despite being approximately 97% identical at the nucleotide level to the other ZEBOV variants^14^,^15^. This apparent phenotypic difference between the variants prompted us to test the action of disinfectants against each of them individually.

Environmental recovery of the Ebola virus uses two metrics, titer of infectious virus and quantity of vRNA to determine virus presence. Field studies predominately focus on measuring vRNA by quantitative RT-PCR (qRT-PCR) to detect Ebola-contaminated surfaces. Such a measure may be misleading as vRNA detection cannot be used to confirm recovery of infectious virus particles and time of disinfection to time of sampling varies greatly. In addition, a few studies have demonstrated that genetic material is recovered much more efficiently than infectious virus particles for Ebola^8^,^16^ and African swine fever^17^ viruses. These biases suggest that vRNA can potentially be detected in the absence of infectious virus, making tissue culture infection a better standard for disinfection efficacy.

This study examined the disinfection of Makona compared to the other two major ZEBOV outbreak variants, Mayinga and Kikwit with common chemical disinfectants that are recommended by the World Health Organization (WHO) for contaminated-surface and hand cleaning during Ebola outbreaks^2^ at average West African temperature. The action of the disinfectants was assessed by infectious virus recovery and compared to vRNA detection for overall quantification efficacy.

## Results

To identify if the Makona variant characteristically differs with respect to decontamination, Makona was compared with the other major Ebola virus outbreak variants. The time-controlled action of 70% ethanol (EtOH) and 0.05%, 0.1%, 0.5% and 1% v/v concentrations of sodium hypochlorite (NaOCl) was tested against dried mixtures of organic soil load and ZEBOV variants on stainless steel carriers at 27 °C/30% RH. The organic soil load mixture mimics bodily excreta, similar to a severe EVD patient^18^,^19^. Standard kill curves were generated for each variant to define disinfection as a whole. The viral variants were then compared against each disinfectant at varying concentrations to understand characteristic differences in response to chemical treatment.

### Standard Kill Curves

**Figure 1:**
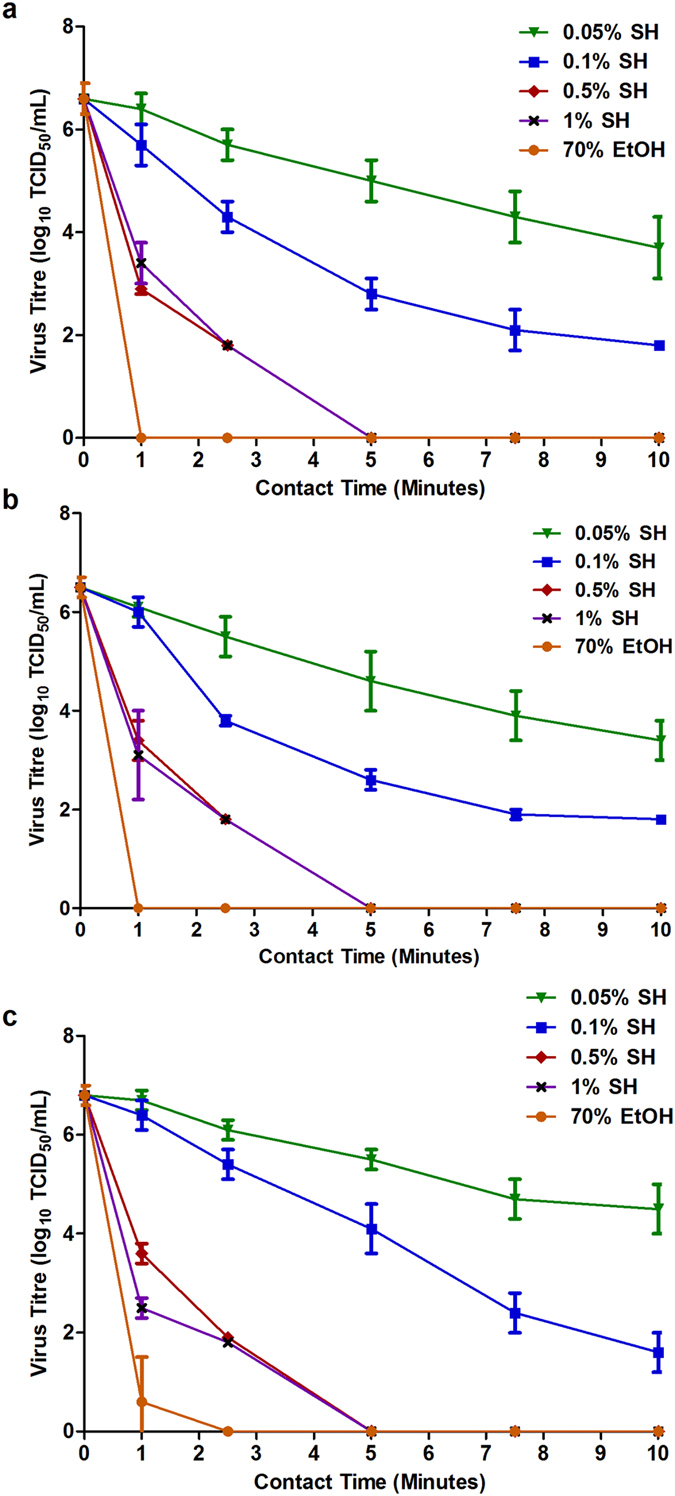
Standard Ebola Kill Curves. Reduction in Ebola virus titres when the (**a**) Mayinga, (**b**) Kikwit and (**c**) Makona variants were treated with sodium hypochlorite (NaOCl) concentrations: 0.05% (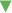), 0.1% (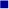), 0.5% (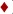) and 1% (**x**) and 70% ethanol (EtOH) (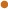) over time. This amalgamated data is from each disinfectant experiment with virus titres from three independent experiments each with three technical replicates is presented.

Upon drying, the titre of the untreated (time point 0), input virus per carrier was determined prior to the addition of disinfectants as 6.6 (+/−0.3) for Mayinga, 6.5 (+/−0.2) for Kikwit and 6.8 (+/−0.2) log_10_ TCID_50_/mL for Makona. The disinfection of all variants followed similar trends. The efficacy of the disinfectants positively correlated to increased concentration and contact time (Fig. 1a,b and c). These analogous responses allowed for the pairwise comparison of Makona with Mayinga and Kikwit for each disinfectant by concentration and time tested.

### The Disinfection of Makona Compared to Previous Outbreak Variants

**Figure 2:**
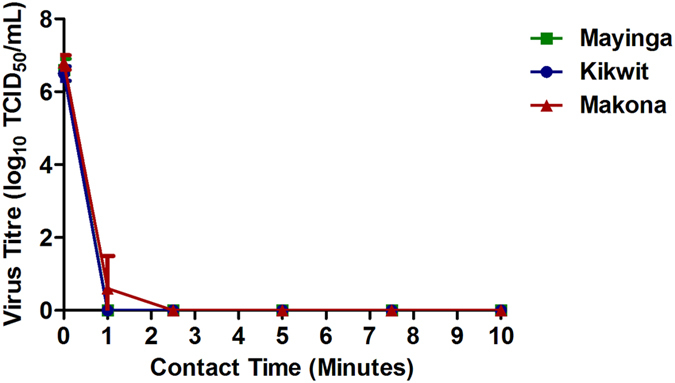
Disinfection of Ebola Virus Variants With 70% Ethanol (EtOH). The efficacy of 70% EtOH over time against the Ebola virus variants Mayinga (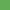), Kikwit (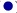) and Makona (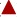) on stainless steel were determined by recovery of infectious particles from the carriers and subsequent quantification using the TCID_50_ assay performed in quintuplicate for each dilution. Virus titres from three independent experiments each with three technical replicates are presented. For statistical analysis, Makona was compared to Mayinga (green) or Kikwit (blue) at the respective time point.

When treated with the single concentration of EtOH (70%) at contact times of 1 through 10 min, Mayinga and Kikwit variants were fully inactivated by 1 min of contact time (Fig. 2). However, detectable titres of Makona survived after this time point in 2/9 replicates, giving an average titre of 0.6 (+/−0.9) log_10_ TCID_50_/mL at 1 min disinfection time. These differences were determined to be not significant by one-way ANOVA with Holm-Bonferroni post-hoc analysis. Infectious virus was not detected between 2.5 to 10 min of contact time with disinfectant (Fig. 2).

**Figure 3:**
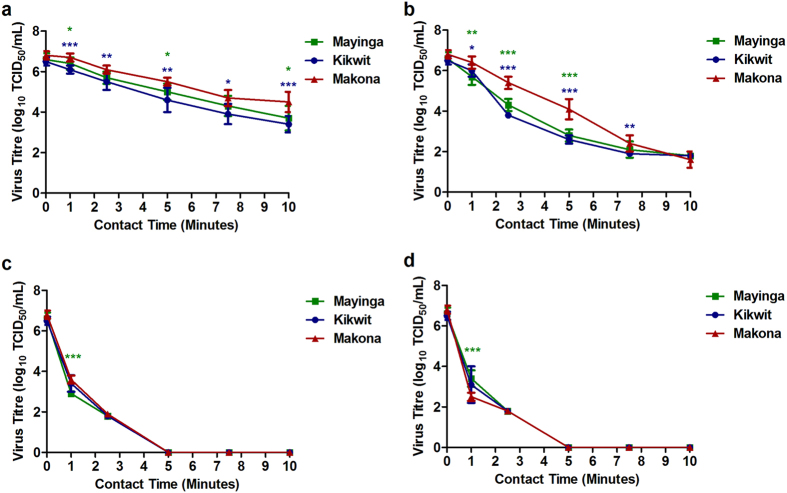
Disinfection of Ebola Virus Variants With Sodium Hypochlorite (NaOCl). The efficacy of NaOCl concentrations: (**a**) 0.05% NaOCl, (**b**) 0.1% NaOCl, (**c**) 0.5% NaOCl, and (**d**) 1% NaOCl, over time against the Ebola virus variants on stainless steel was assessed by measuring infectious particle recovery and titration by TCID_50_ assay performed in quintuplicate for each dilution. Virus titres from three independent experiments each with three technical replicates are presented. For statistical analysis, Makona (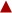) was compared to Mayinga (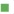), Kikwit (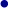) at the respective time point, *p < 0.05, **p < 0.01, ***p < 0.001.

As shown in the standard kill curves (Fig. 1a,b and c), the variants demonstrated concentration-dependent disinfection when treated with NaOCl. The more diluted solutions did not completely inactivate the variants. Over three log_10_ TCID_50_/mL of virus was recovered for each virus: 3.7 (Mayinga), 3.4 (Kikwit) and 4.5 (Makona) treated with 0.05% NaOCl (Fig. 3a). Less virus was detected at the 0.1% dilution: 1.8 (Mayinga), 1.8 (Kikwit) and 1.6 (Makona) log_10_ TCID_50_/mL (Fig. 3b) remained after 10 min of contact. The more concentrated NaOCl solutions (0.5% and 1%) enabled strong reductions in titres, as virus was not detectable by 5 min of contact (Fig. 3c and Fig. 3d).

The weakest concentration of NaOCl (0.05%) revealed differences in Makona viability when compared to both Mayinga and Kikwit. Using a time-specific approach to compare the quantity of Makona to both Mayinga and Kikwit remaining after treatment, Makona had statistically significantly higher titres remaining after treatment, expressed as log_10_ TCID_50_/mL, then Mayinga at the specified contact times of 1 min (0.3 log_10_, p < 0.05), 5 min (0.5 log_10_, p < 0.05) and 10 min (0.8 log_10_, p < 0.05). This pattern was also evident for Makona versus Kikwit at 1 min (0.6 log_10_, p < 0.001), 2.5 min (0.6 log_10_, p < 0.01), 5 min (0.9 log_10_, p < 0.01), 7.5 min (0.8 log_10_, p < 0.05) and 10 min (1.1 log_10_, p < 0.001) (Fig. 3a). A stronger solution of 0.1% NaOCl also indicated that Makona was hardier than Mayinga and Kikwit. Statistically significant titre differences were apparent as Makona had higher titres after treatment at the contact times than Mayinga: 1 min (0.7 log_10_, p < 0.01), 2.5 min (1.1 log_10_, p < 0.001) and 5 min (1.3 log_10_, p < 0.001). Similar trends in higher post-treatment titres were observed at the time points of 1 min (0.4 log_10_, p < 0.05), 2.5 min (1.6 log_10_, p < 0.001), 5 min (1.5 log_10_, p < 0.001) and 7.5 min (0.5 log_10_, p < 0.01) in comparison of Makona to Kikwit (Fig. 3b).

Evaluation of residual titre is done by serial dilutions of treated samples from undiluted (10^0^) to 10^−7^, and viral replication is assessed by the appearance of cytopathic effect (CPE). However, it is important to note that CPE caused by the variants were only visible at the 10^−1^ dilution, as the undiluted (10^0^) samples which contained the combination of 0.5% or 1% NaOCl and its neutralizer (1% sodium thiosulfate) is cytotoxic to cell monolayers and this complicated the observation of CPE. The absence of virus from 10^−1^ to 10^−7^ would suggest a complete kill had occurred. Thus to confirm complete kill, flasks of VeroE6 cells were infected by pooling the triplicate undiluted (10^0^) samples from each biological replicate, and incubated for 14 days to await CPE development. The absence of CPE indicated that the 10^0^ dilution was negative for virus (defined as complete kill) while the presence of CPE indicated that 10^0^ was indeed positive for virus (calculated as 1.8 log_10_), as the combination of 0.5% or 1% NaOCl with 1% sodium thiosulfate did not display any signs of cytotoxicity (Table 1). This scheme evaluated the undiluted samples for the 2.5 to 10 min time points, as CPE was not visible beyond 10^0^ for 0.5% and 1% NaOCl. Overall, the higher concentrations of NaOCl (0.5% and 1%) resulted in drastic reductions in Ebola virus titres for all variants. When 0.5% NaOCl was applied, the titre differences were subtle: at 1 min (0.7 log_10_, p < 0.001) and 2.5 min (0.1 log_10_, not significant) Makona versus Mayinga and, at 1 min (0.2, not significant) and 2.5 min (0.1 log_10_, not significant) Makona against Kikwit. The infection of VeroE6 cells in flasks revealed that all of the variants were inactivated by 5 min of contact (Fig. 3c) (Table 1). Interestingly Makona appeared to be more susceptible to 1% NaOCl than both Mayinga and Kikwit. As the amounts of Mayinga and Kikwit remaining after 1 min was higher than Makona, 0.9 log_10_ (p < 0.001) and 0.6 log_10_ (not significant), respectively. At 2.5 min, virus culture in flasks of VeroE6 cells indicated that virus was present at 10^0^ for Mayinga, Kikwit and Makona, calculated as 1.8 log_10_ but, not viable after 5 min of contact (Fig. 3d) (Table 1).

### The Reliability of Ebola RNA as a Measurement of Disinfection Success

In order to uncover the utility of Ebola virus RNA detection as a representation of infectious virus particles, we performed qRT-PCR on vRNA isolated after disinfectant treatment at each contact time. The preliminary data suggests that 70% EtOH and high concentrations of NaOCl inactivate infectious virus particles but have little effect on vRNA quantification by qRT-PCR. The Ebola vRNA extracted from the pooled triplicate samples of singular biological replicates from Mayinga and Makona against 70% EtOH, 0.5% and 1% NaOCl was measured in cycle threshold (Ct) and genome equivalents (GE)/mL.

For comparison purposes, only the time points which vRNA was recovered without the presence of infectious virus particles is described versus the untreated virus controls (0 min). These controls demonstrated an average Ct of 11 (+/−1) and 11 (+/−2), and an average GE of 12 (+/−0.3) log_10_/mL and 12 (+/−0.4) log_10_/mL (Table 2). These values correspond to virus titres of 6.6 (+/−0.3) log_10_ TCID_50_/mL for Mayinga and 6.8 (+/−0.2) log_10_ TCID_50_/mL for Makona. After treatment with 70% EtOH, vRNA was detected at 1 min (15 and 10 Ct), 2.5 (13 and 10 Ct), 5 min (15 and 11 Ct), 7.5 min (16 and 9 Ct) and 10 min (15 and 11 Ct) (Table 2). The 1 min time point was included for Makona as most replicates did not have infectious particles detected, thus being similar to what was observed for both Mayinga and Kikwit. These Ct values resulted in GE values ranging from 10 to 12 log_10_/mL (Table 2).

The comparison for treatment with ≥0.5% NaOCl resulting in vRNA without infectious particles for Mayinga and Makona were analyzed next. Beginning with 0.5% NaOCl, Ct values at the respective contact times were: 5 min (17 and 19 Ct), 7.5 min (18 and 18 Ct) and 10 min (19 and 20 Ct) (Table 2) and these corresponded to GE ranging from 9 to 10 log_10_/mL (Table 2). Similarly for 1% NaOCl, the reductions in detectable RNA for Mayinga and Makona were apparent at 5 min (19 and 22 Ct), 7.5 min (20 and 27 Ct) and 10 min (21 and 35 Ct) of contact with 1% NaOCl (Table 2), resulting in GE within 4 to 9 log_10_/mL (Table 2).

## Discussion

Disinfection of most major Ebola virus outbreak variants was achieved after 1 min of contact with 70% EtOH. Makona was an exception and required 2.5 min. Strong solutions of NaOCl (≥0.5%) inactivated the variants by 5 min of contact whereas, weaker concentrations of NaOCl (0.05% and 0.1%) were not able to fully inactivate any of the variants. Comparing the variants revealed that Makona was less susceptible than Mayinga and Kikwit to 0.05%, 0.1% NaOCl and 70% EtOH, but differences were not observed with higher concentrations of disinfectant. The discovery of high amounts of vRNA remaining after 70% EtOH and ≥0.5% NaOCl treatment suggested that genetic detection may not be a suitable methodology for accurately measuring disinfection as infectious virus may no longer be present.

The United States Centers for Disease Control and Prevention (CDC) provides instructions for the daily preparation of the WHO recommended 0.5% chlorine for surface cleaning and 0.05% chlorine for hand washing used during Ebola virus outbreaks^2^,^20^. The NaOCl solutions used in this study were prepared following the CDC guideline. A recent study identified that NaOCl concentrations mixed with water (called stabilized NaOCl), remained stable for up to 30 days when stored at 25–35 °C^21^. In our solutions, we used hard water (distilled water with 0.04% w/v calcium carbonate) to better reflect water impurities in an outbreak scenario. The stability of NaOCl “household bleach” is great news for local populations in need of disinfecting their homes or for doctors in ETCs. Although other forms of chlorine-based solutions are available for use in ETCs, our study only investigated NaOCl as it is readily available for use in laboratories, households and hospitals.

While the genomes of the ZEBOV variants are 97% identical at the nucleotide level^14^, there are 80 and 76 respective non-synonymous alterations in the genomes of Mayinga and Kikwit compared to Makona^15^. The majority are present within the nucleoprotein (NP), the glycoprotein (GP) and the RNA-dependent RNA polymerase (L) coding sequences^15^. It is unclear whether these amino acid changes were responsible for the apparent decreased susceptibility of Makona against 70% EtOH, and 0.05% and 0.1% NaOCl. Perhaps the amino acid changes in Makona lead to differences in receptor binding/replication in VeroE6 cells or increase virion structure stability during treatment with the disinfectants. Regardless of this speculation, the WHO and CDC-recommended concentration of NaOCl (0.5%) is suitable for complete killing of Ebola virus at 5 min. However, there remains cause for concern regarding the doffing of personal protective equipment (PPE) for medical staff that are leaving an Ebola treatment zone, as the bare-hand washing stage uses 0.05% NaOCl for a 40–60 second contact time (or until dry)^2^. Even though our data suggest that 0.05% NaOCl would not provide adequate disinfection during un-gloved hand-washing, a stronger chlorine solution would result in skin irritation or burns^22^. Although it is reasonable to assume the scrubbing action during hand washing could sufficiently remove or rinse away Ebola-contamination, we should consider alternatives to 0.05% NaOCl until empirical data is generated supporting the currently acted practice of using 0.05% NaOCl. Perhaps alcohol-based (70% EtOH) as suggested by the WHO^2^ or povidone-iodine based solutions^23^ should be considered as alternatives as both have shown rapid inactivation of Ebola virus. It is noteworthy that although 70% EtOH is efficient at inactivating Ebola after 1 min of contact, the Makona variant was still viable on 2/9 replicates and in our previously generated data, on 3/9 replicates^9^. Combined, these data suggest that Makona may be slightly more resistant than Mayinga and Kikwit, although this outcome is not statistically significant. Regardless, 70% EtOH would be better in place of 0.05% NaOCl for hand washing with respect to killing action.

The lack of connection between Ebola virus particles and the amount of vRNA quantified after disinfection highlights the issue of using vRNA as a sole measure of decontamination success. Youkee *et al*., endorse the use of qRT-PCR to demonstrate decontamination efficacy in Ebola treatment units. These authors describe the detection of Ebola vRNA from swabbing performed at 30 and 60 min after cleansing with 0.5% NaOCl, thereby suggesting a nosocomial risk. Although interesting, it is noteworthy that attempts to culture Ebola virus from these swabs was not possible due to lack of suitable facility and containment resources at the treatment units, and nearly one-third of the swab sample-replicates were actually positive for vRNA with Ct values ranging from 32–39 (40 Ct was considered negative)^24^. Another study which performed vRNA and culturing from swabs showed that despite routine daily cleaning with 0.5% NaOCl, only vRNA, and not culturable virus, was obtained from swabbing blood-contaminated floor areas, six days after an Ebola-infected patient was discharged from an Italian hospital^25^. This result is similar to the swabbing of blood-contaminated locations in a Ugandan isolation ward in which virus cultures were negative, despite the samples testing positive for vRNA^26^. The lack of connection between vRNA detection and, the inability to culture virus was evident in blood samples obtained from Ebola-infected patients concurrently treated with immuno-therapeutics. Spengler *et al*., describe virus isolation by culturing was not possible in patients after 12 days of symptom onset or with Ct > 35.5, although this was also the case in some patients that had Ct values < 35.5^27^. It is unclear whether the antibody-based therapies or the adaptive immune responses complicated the ability to isolate infectious virions from blood samples. The comparison between infectious virus and vRNA in our study show that qRT-PCR can be misleading as high Ct values corresponding to significant levels of vRNA were obtained from disinfectant-inactivated virus particles. This is especially striking, considering EtOH does not negatively impact vRNA^28^ and while NaOCl at concentrations of 0.5% and 1% significantly reduce vRNA levels, it may not be fully eliminated^28^,^29^,^30^. Furthermore, primer/probe sets only require small regions of the genome (NP or L) for signal production, thus full-length RNA and infectious particles may not even be present. Therefore, despite RNA detection serving as a useful tool in areas without a high containment laboratory for culturing, one must be cautious of making the extension from detection of vRNA in the environment to the presence of infectious virus particles. More Research is necessary to provide the link between infectious virus stability and vRNA, especially in the absence of virus culturing.

The data presented indicates that the major Ebola outbreak variants can be efficiently inactivated with 70% EtOH by 2.5 min and ≥0.5% NaOCl at 5 min. Concentrations of NaOCl that are suboptimal for complete inactivation demonstrated that Makona was more resistant to disinfection than Mayinga and Kikwit. Performing qRT-PCR on inactivated Ebola samples demonstrated that high amounts of vRNA remained despite complete killing of infectious particles. Evaluation of effective decontamination should be determined or corroborated by assays that quantify infectious virus and not solely by the detection of viral components.

## Methods

### Cells, viruses and chemicals

#### Cells

African green monkey kidney (VeroE6) cells (ATCC CRL-1586) were propagated in growth media consisting of Dulbecco’s modified eagle medium (DMEM) (HyClone) which included 10% Fetal Bovine Serum (FBS) (Gibco) and 1% antibiotics (Penicillin-Streptomycin) (Gibco) at 37 °C/5% CO_2_. For virus culture, virus maintenance medium was the same as growth medium with FBS reduced to 2%.

#### Viruses

Ebola virus variant stocks of 1976 Mayinga, 1995 Kikwit and 2014 Makona (C05) were cultivated in VeroE6 cells within the Containment Level 4 (CL-4) laboratory at the National Microbiology Laboratory (NML) in the Canadian Science Centre for Human and Animal Health (CSCHAH) located in Winnipeg, Canada. Virus-containing supernatants were harvested at approximately 80% CPE (10–12 days post-infection). Virus samples were clarified by centrifugation at 5000 × g for 10 min followed by centrifugation through a 20% sucrose cushion for 2 hours at 108,000 × g to pellet the virus. Virus pellets were resuspended in virus maintenance medium and stored at −80 °C.

#### Chemicals

Sodium hypochlorite (NaOCl) from 10.8% stock concentration (Imperial Soap and Supplies Limited) or ethanol (EtOH) from 95% stock concentration (Commercial Alcohols) were diluted, volume/volume into hard water (distilled water with 440 ppm calcium carbonate) prior to each assay. The neutralizers for the NaOCl and EtOH disinfectants were prepared prior to each assay as follows; 1% sodium thiosulfate (from 10% stock concentrations) mixed volume/volume with DMEM or solely DMEM, respectively.

### Ebola Virus Disinfection

As summarized previously^9^,^18^ the quantitative carrier test-two (QCT-2) (ASTM E-2197) was utilized for the disinfection experiments. Briefly, 80 μl of organic soil load meant to mimic residual bodily fluids and comprised of: 12.5 μL 5% bovine serum albumin (Sigma), 17.5 μL 5% tryptone (Becton Dickinson) and 50 μL 0.4% bovine mucin (Sigma) was mixed with 125 μl of specified virus variant: Mayinga (passage 3), Kikwit (passage 3) and Makona (passage 2). The soil load-virus mixture was deposited (10 μl) onto disk-shaped stainless steel carriers and allowed to dry for 1 hour at 27 °C (simulating tropical temperature) on a Boekel Scientific Slide Moat incubator/hot plate (Fisher Scientific) inside of a biological safety cabinet. Once air-dried, the carriers from each treatment time point and the untreated (time point 0) control was transferred into a well of a 12-well plate before being subjected to disinfection. Disinfectant was pre-heated to 27 °C and 50 μl was placed onto the dried inoculum/carrier and incubated for the contact times (1, 2.5, 5, 7.5 or 10 min). Cessation of disinfectant activity was achieved by the addition of a neutralizer (950 μl for treated samples). For control purposes, 990 μl of the neutralizer was also added to the untreated controls (time point 0). Virus elution immediately followed via washing/scrapping of the carriers, 500 μl of the 1 mL eluate was used for fifty percent tissue culture infectious dose (TCID_50_) quantification. For 0.5% and 1% NaOCl, the other 500 μl from each triplicate sample were pooled together and used for tissue culture flask infection (complete kill confirmation) and for quantitative reverse transcription-polymerase chain reaction (qRT-PCR) analysis. Disinfection experiments were performed independently as triplicate experiments each with three technical repetitions.

### Cytotoxicity and complete kill confirmation

The combination of 0.5% or 1% NaOCl concentrations with the 1% sodium thiosulfate neutralizer are toxic to VeroE6 cells only in undiluted (10^0^) samples during virus absorption. To remedy this, the remaining 500 μl of undiluted sample from each replicate were pooled together and used to infect T150 cell culture flasks of VeroE6 cells. After virus adsorption for 1 hour at 37 °C, virus maintenance medium was added making a final volume of 30 mL, flasks were incubated at 37 °C. Monolayers were visually analyzed for signs of CPE and cytotoxicity on 7 and 14 days post-infection.

### Fifty Percent Tissue Culture Infection Dose (TCID_50_) Assay

VeroE6 cells were prepared for 80% confluency in 96 well plates 24 hours prior to assay. After virus elution from stainless steel carriers, 500 μl of this supernatant was titrated (10-fold serial dilution) and 50 μl of each dilution was added to VeroE6 monolayers in 96-well culture plates in quintuplicate for 1 hour at 37 °C/5% CO_2_. Then, following the addition of 150 μl of virus maintenance media, the cells were incubated at 37 °C/5% CO_2_ and were scored for CPE on 7 and 14 days post-infection. Titres as TCID_50_/mL were determined using the Reed and Muench formula^31^.

### Quantitative reverse transcription-polymerase chain reaction (qRT-PCR)

After virus elution from carriers treated with 70% EtOH and ≥0.5% NaOCl, the triplicate samples from a singular biological experiment were pooled together. The vRNA was extracted (Qiagen) from 140 μl from the pooled supernatants and was quantified by qRT-PCR using Light Cycler 480 RNA Master Hydrolysis (Roche) and with the following ZEBOV primer/probe set: 300 nm of ZEBOV LF (CAGCCAGCAATTTCTTCCAT) and ZEBOV LR (TTTCGGTTGCTGTTTCTGTG) primers mixed with 150 nm of ZEBOV LP1 (FAM-ATCATTGGCGTACTGGAGGAGCAG) and ZEBOV LP2 (FAM-TCATTGGCGTACTGGAGGAGCAGG) probe combination. Data analysis and genome equivalents were quantified from a standard curve obtained from Ebo-Rib (Ebola reverse genetics system) DNA plasmid^32^.

### Statistical Analysis

Infectious virus particles as measured by TCID_50_ assay were compared for Makona against Mayinga and Makona against Kikwit using one-way ANOVA and Holm-Bonferroni post-hoc analysis using Excel to determine P values of <0.05, <0.01 and <0.001.

## Additional Information

**How to cite this article**: Cook, B. W. M. *et al*. The Disinfection Characteristics of Ebola Virus Outbreak Variants. *Sci. Rep.* **6**, 38293; doi: 10.1038/srep38293 (2016).

**Publisher’s note:** Springer Nature remains neutral with regard to jurisdictional claims in published maps and institutional affiliations.

## Figures and Tables

**Table 1:** Complete Kill of Ebola Virus Variants.

Treatment	Time Point	CPE (Mayinga/Kikwit/Makona)	Cytotoxicity
0.5% Sodium Hypochlorite (NaOCl)	0 Minute N = 3 (R1)	+/+/+	−/−/−
	0 Minute N = 3 (R2)	+/+/+	−/−/−
	0 Minute N = 3 (R3)	+/+/+	−/−/−
	1 Minute N = 3 (R1)	+/+/+	−/−/−
	1 Minute N = 3 (R2)	+/+/+	−/−/−
	1 Minute N = 3 (R3)	+/+/+	−/−/−
	2.5 Minutes N = 3 (R1)	+/−/+	−/−/−
	2.5 Minutes N = 3 (R2)	−/+/−	−/−/−
	2.5 Minutes N = 3 (R3)	+/+/+	−/−/−
	5 Minutes N = 3 (R1)	−/−/−	−/−/−
	5 Minutes N = 3 (R2)	−/−/−	−/−/−
	5 Minutes N = 3 (R3)	−/−/−	−/−/−
	7.5 Minutes N = 3 (R1)	−/−/−	−/−/−
	7.5 Minutes N = 3 (R2)	−/−/−	−/−/−
	7.5 Minutes N = 3 (R3)	−/−/−	−/−/−
	10 Minutes N = 3 (R1)	−/−/−	−/−/−
	10 Minutes N = 3 (R2)	−/−/−	−/−/−
	10 Minutes N = 3 (R3)	−/−/−	−/−/−
1% Sodium Hypochlorite (NaOCl)	0 Minute N = 3 (R1)	+/+/+	−/−/−
	0 Minute N = 3 (R2)	+/+/+	−/−/−
	0 Minute N = 3 (R3)	+/+/+	−/−/−
	1 Minute N = 3 (R1)	+/+/+	−/−/−
	1 Minute N = 3 (R2)	+/+/+	−/−/−
	1 Minute N = 3 (R3)	+/+/+	−/−/−
	2.5 Minutes N = 3 (R1)	+/+/+	−/−/−
	2.5 Minutes N = 3 (R2)	−/+/+	−/−/−
	2.5 Minutes N = 3 (R3)	+/+/+	−/−/−
	5 Minutes N = 3 (R1)	−/−/−	−/−/−
	5 Minutes N = 3 (R2)	−/−/−	−/−/−
	5 Minutes N = 3 (R3)	−/−/−	−/−/−
	7.5 Minutes N = 3 (R1)	−/−/−	−/−/−
	7.5 Minutes N = 3 (R2)	−/−/−	−/−/−
	7.5 Minutes N = 3 (R3)	−/−/−	−/−/−
	10 Minutes N = 3 (R1)	−/−/−	−/−/−
	10 Minutes N = 3 (R2)	−/−/−	−/−/−
	10 Minutes N = 3 (R3)	−/−/−	−/−/−

**Table 2:** Ebola RNA Recovery From Disinfectant-Inactivated Ebola Virus Variants.

Disinfectant		70% EtOH^a^		0.5% NaOCl^b^		1% NaOCl^b^	
Variants	Contact Time (Min)	Ct^c^	GE/mL^d^	Ct^c^	GE/mL^d^	Ct^c^	GE/mL^d^
Mayinga	0	11	12	11	12	11	12
Makona	0	11	12	11	12	11	12
Mayinga	1	15	10	16	10	15	11
Makona	1	10	12	14	11	16	10
Mayinga	2.5	13	11	15	10	17	10
Makona	2.5	10	12	16	10	19	9
Mayinga	5	15	10	17	10	19	9
Makona	5	11	11	19	9	22	8
Mayinga	7.5	16	10	18	9	20	9
Makona	7.5	9	12	18	9	27	7
Mayinga	10	15	10	19	9	21	9
Makona	10	11	12	20	9	35	4

^a^EtOH = ethanol.

^b^NaOCl = Sodium hypochlorite.

^c^Ct = cycle threshold.

^d^GE/mL = Genome equivalents per mL presented in Log_10_ scale.
